# Broiler farming and antibiotic use through an agency theory lens. A case study from West Bengal, India

**DOI:** 10.1371/journal.pone.0314090

**Published:** 2025-01-09

**Authors:** Mathew Hennessey, Indranil Samanta, Guillaume Fournié, Matthew Quaife, Meenakshi Gautham, Haidaruliman Paleja, Kumaravel Papaiyan, Ripan Biswas, Pablo Alarcon

**Affiliations:** 1 Veterinary Epidemiology, Economics and Public Health Group, Department of Pathobiology and Population Sciences, WOAH Collaborating Centre in Risk Analysis and Modelling, Royal Veterinary College, London, United Kingdom; 2 Department of Veterinary Microbiology, West Bengal University of Animal and Fishery Sciences, Kolkata, India; 3 INRAE, VetAgro Sup, UMR EPIA, Université de Lyon, Marcy l′Etoile, France; 4 INRAE, VetAgro Sup, UMR EPIA, Université Clermont Auvergne, Saint Genes Champanelle, France; 5 Evidera, United Kingdom; 6 Department of Global Health and Development, Faculty of Public Health and Policy, London School of Hygiene and Tropical Medicine, London, United Kingdom; 7 Department of Veterinary Biotechnology, College of Veterinary Science and Animal Husbandry, Kamdhenu University, Anand, India; 8 Veterinary College and Research Institute, TANUVAS, Udumalpet, India; 9 Department of Veterinary Public Health, West Bengal University of Animal and Fishery Sciences, Kolkata, India; Ain Shams University Faculty of Agriculture, EGYPT

## Abstract

Chicken meat (broiler) production is a rapidly growing livestock sector in India, and one dominated by contract farming. Studies have reported high levels of antibiotic use in Indian broiler farms which is concerning given this is one of the driving forces for the development of antibiotic resistance. This study used the economic lens of agency theory to examine strategic decisions which occur during contract broiler production and their potential impact on antibiotic use, using West Bengal as a case study. Agency theory focuses on the informational asymmetry and opportunism between service providers and seekers and the subsequent agency cost needed to avoid aberrant outcomes. Interviews were conducted with key informants (n = 6) and stakeholders (n = 20) associated with broiler production, and broiler farmers (17 contract and four non-contract), using online and face-to-face interviews. Data were analysed descriptively using manifest content analysis and interpretatively using reflexive thematic analysis. Contract farming in West Bengal exists within a series of inter-dependent relationships, many of which contain information asymmetry and can be subject to opportunism. Positioning contract companies as principals seeking labour from agents, we see how out-sourcing of production to distal farms led to antibiotics being used as a risk mitigation strategy. This was further compounded by concerns about the *Mycoplasma* status of breeding stock, and a perception that broiler day old chicks were infected, resulting in use of antibiotics belonging to classes deemed critically important for human health. While antibiotic use decisions were predominately made by contract companies, they were dependent on the decisions farmers and breeding companies made concerning biosecurity and production practices. In turn, farmers’ decisions were shaped by factors such as access to financial and social capital. Thus, efforts to reduce antibiotic use in West Bengal’s broilers must not just focus on changing the prescribing behavior of individuals but more broadly consider the environment within which contracting exists.

## 1. Introduction

Intensive chicken meat (broiler) production is one of the most rapidly growing livestock sectors, both globally and within India [1, 2] fueled by universal acceptance among meat eaters [3], short production cycles, and relatively low food conversion ratios [4]. However, studies have reported high levels of antibiotic use in Indian broiler production, including use of antibiotics deemed critically important for human health [5–8]. This is concerning given antibiotic use is one of the driving forces for the development of antibiotic resistance [9–13] a threat expected to have enormous social and economic implications, in India and globally, if not effectively controlled [14].

Before 1965, when Srinivasa Farms established the first intensive commercial poultry farm in India [15], production mainly occurred in extensive ‘backyard’ systems. Here, various indigenous breeds were raised for eggs and meat in free-range, semi-scavenging, and small-scale commercial systems [16]. However, after the mid-1980s India experienced rapid growth in the commercial broiler sector [17] particularly in the states of Tamil Nadu, Karnataka, Andhra Pradesh, Maharashtra, and West Benga, a period known as India’s poultry revolution [2].

Broiler production can occur under a range of conditions, independent production, varying contract arrangements [18, 19], and vertically integrated production. During independent production, farmers source and finance poultry inputs themselves, manage production, and arrange broiler sales by interacting with other stakeholders. Under formal production marketing contracts, companies recruit farmers, using their land, farm infrastructure, and labour to produce broilers. Here, most inputs and production decisions come from the contracting company, which interacts with farmers through company representatives (branch managers and supervisors). At the end of production birds are collected from farms and farmers paid a fee per finished bird [2, 20]. In formal input marketing contracts, poultry companies supply inputs (e.g., chicks or feed) to intermediary agents who then supply inputs to farmers, often through credit, via informal output marketing contracts. These agents, and other traders and middlemen, then arrange for broilers to be distributed to the market. Alternatively, some poultry companies are involved with vertically integrated broiler production where birds are raised, slaughtered, and processed on their own premises, i.e., all value chain activities are controlled by the company.

Within the Indian poultry industry, it has been estimated that 70% of antibiotics are used prophylactically or for growth promotion [6], with higher levels of antibiotic usage in broiler chickens compared to layers [5]. Until recently, it was reported that colistin, a last resort antibiotic for human health, was being sold without a prescription for poultry growth promotion [5, 21]. However, a general ban on the manufacture and sale of colistin was implemented by the Ministry of Health in 2019 [22]. India’s 2017 National Action Plan on antimicrobial resistance contains a strategic priority focusing on improving the use of antimicrobials in livestock through regulation, surveillance, and stewardship [23]. The plan provides some explicit guidelines for how antibiotic use can be refined, including 1) restricting and phasing out of antibiotic growth promoters and prophylaxis in animals, 2) developing evidence-based local treatment guidelines for food animals, and 3) restricting and phasing out the use of critically important antibiotics for humans in food animals. Thus, there is evidence to suggest that antibiotics are being used in Indian broiler production in ways which do not align with India’s national plans to mitigate the risk of antimicrobial resistance.

Given global and national concerns over antibiotic use and resistance, the aim of this study was to investigate stakeholder relationships during contract broiler production which impact antibiotic use, using West Bengal as a case study. The specific objectives were to; 1) describe the characteristics of broiler production including antibiotic use, 2) identify major themes using the lens of agency theory, and 3) identify key strategic decisions impacting antibiotic use. It was anticipated that these findings would have relevance for future stewardship interventions to improve antibiotic use, a topic we engage with in the discussion of this paper.

## 2. Methods

### 2.1 Theoretical framing

Contract relationships are typically examined under two branches of neoclassical economics, agency theory deriving from rational choice theory [24, 25] and transaction cost economics stemming from organisational theory [26]. Modern agency theory is considered an amalgamation of Ross’ [25] economic theory of agency and Mitnick’s [24] institutional theory of agency [27]. Both have their origins in the ‘theory of the firm’ [28], a group of neo-classical economic theories that explain the nature of firms concerning the market. Ross’ [25] theory described the incentive structures needed to align activities within the relationship. Mitnick’s [24] theory focused on how institutions form in response to agency problems, i.e., a descriptive theory explaining the context of agency. Transaction cost economics originated from the new institutional economic framework which examined costs associated with the trading of goods and services. Williamson [26] described transaction costs as “the costs of negotiating, establishing, safeguarding and enforcing a contractual agreement” and the framework also considers the governance structures within which contracting occurs.

Agency theory can be used to examine contract relationships between actors seeking and providing a service by examining three main areas of interest; information asymmetry, opportunism, and agency cost/incentives (Table 1). The nature of acquisition-provision relationships creates potential for information asymmetry, with service providers having information that service seekers do not. Consequently, actors can engage in opportunistic behaviours as the expense of others. This may occur when information asymmetry exists prior to the contract being set out (ex-ante adverse selection) or after the contract (ex-post moral hazard). Subsequently, agency theory examines the types of agency costs/incentives needed to avoid aberrant outcomes.

**Table 1:** Agency theory framework.

Category	Description	Examples from animal healthcare literature
Information asymmetry	When hidden information exists between contracting parties, e.g., when effort levels cannot be observed, or the services provided are beyond the technical comprehension of the seeker	Meat traders are not privy to which muscle farmers use to inject their cattle [34]
Opportunism	*Adverse selection*:	
	Ex-ante opportunism occurs when information asymmetry exists prior to the contract being set out, for example producing lower quality products in markets which lack quality price differentials or adoption of insurance by higher risk individuals	Establishing varying premiums according to pet health risk is necessary to prevent insurance markets from collapsing [38]
	*Moral hazard*:	
	Ex-post opportunism occurs when information asymmetry exists after the contract has been set out, for example a provider putting in less effort to the contract arrangement to benefit themselves	Farmers may choose to inject in rump muscles for ease at the expense of carcass quality [34]
Agency cost/ incentives	Costs that the service seeker must expend in order to align the behaviour of the provider with their interests, for example contracts which contain performance related bonuses	Meat traders need to invest in traceability systems to persuade farmers to inject in non-prime muscles [34]

Within livestock production and animal healthcare, agency theory has been used to investigate relationships between; governments and farmers in controlling disease outbreaks [29–32]; livestock traders and purchasers in ensuring commodity quality [33, 34]; and farmers and contract companies concerning the nature of contracts [35–37] (two examples are provided in Table 1).

A previous review of animal healthcare literature, however, found a lack of papers using agency theory in a theoretical or empirical manner with a focus on the poultry sector or antibiotic use [39]. Saha et al. [40] use aspects of agency theory to examine the strategies employed by feed mills in their contract relationships with broiler farmers in Bangladesh, exploring the conditions where farmers accept contracts and abide by their conditions.

Given that contracting is a major part of broiler farming in West Bengal, we elected to explore the potential for agency theory to act as a theoretical lens to investigate strategic decisions leading to antibiotic use. Here, we consider those decisions taken by stakeholders which consider and are impacted by the decisions of other stakeholders. During strategic decision making, data is gathered and processed, analysis of options is conducted, and a choice between alternatives is made [41]. Elbanna [42] describes how strategic decisions are important to the livelihood and survival of organisations. Consequently, strategic decisions are complex and have effects that may be difficult to undo, i.e., they have long-term implications for enterprises [43].

### 2.2 Study setting

West Bengal was chosen as a suitable case study site as it is one of India’s major agricultural states and with an estimated 53 million poultry has the fifth largest poultry population in India (behind Andhra Pradesh, Tamil Nadu, Maharashtra, and Karnataka) [44].

### 2.3 Study design and data collection

We used networks of contacts from an existing research project—the One Health Antibiotic Stewardship in Society (OASIS) project based in West Bengal–to recruit participants during two rounds of data collection using purposive and convenience sampling in order to elicit sufficient variability and multiple perspectives in the responses (Table 2). Two rounds of data collection occurred as COVID-19 restrictions prevented initial fieldwork from taking place.

**Table 2:** Summary of interview participants for the West Bengal case study.

	Participant details	Number
Round 1. Online interviews Key-informants	Veterinarian (academic)	2
	Veterinarian (poultry consultant)	2
	Veterinarian (animal health company technician)	2
Round 2. Fieldwork–stakeholders	Government veterinarians	1
	Contract company branch managers	2
	Contract company general managers	2
	Contract company supervisors	2
	Poultry academic	2
	Poultry consultant	1
	Chick/feed dealer	6
Round 2. Fieldwork—Broiler farmers	Contract broiler farmers	17
	Non-contract broiler farmers	4

An initial online round of interviews took place to provide preliminary data and aid the planning of fieldwork activities. Three pilot interviews were conducted (26^th^ March to 16^th^ April 2021) to test interview questions. An interview guide was constructed based on strategic decision-making concerning antibiotic use. Specifically, we focused on, 1) the type and quantity of antibiotics used in broiler production, 2) the decisions which impact antibiotic use, 3) stakeholders’ perceptions of antibiotic misuse, and 4) antibiotic policy and guidelines (S1 File). In the first round (from 24^th^ April to 5^th^ July 2021) interviews were conducted online (Zoom, MS Teams, WhatsApp) with key-informants, defined as individuals with an anticipated high-level knowledge of the broiler sector. Of the 18 interviews conducted, six participants were based in West Bengal and data from their interviews are included in this analysis. During the online interviews, key-informants expressed they wanted to conduct the interviews in English and so this language was used, with audio recordings lasting between 53 and 82 minutes (mean 65).

During round two, when fieldwork to India was possible (24^th^ February to 29^th^ March 2022), interviews were conducted in person, in and around Kolkata, West Bengal, with poultry farmers and stakeholders. Being a qualitative and exploratory study, it was the intention to interview a range of stakeholders from across the poultry sector and as many contract and non-contract farmers as possible. The interview guide for the second round focused on the following topics, 1) stakeholders’ background leading into broiler production, 2) access to production inputs, 3) labour, income and the nature of contract relationships between input suppliers and producers, and 4) antibiotic use practices (S2 File). Thirty-seven interviews and informal discussions were conducted in English and Bangla, with a co-author (RB) translating between the two languages. Audio recordings (n = 30) lasted between 17 and 77 minutes (mean 36).

During both data collection phases participants were given a consent form and information sheet prior to the interview (S3 & S4 Files) and written consent obtained. Interviews were voice recorded when participants provided consent to do so, otherwise, written notes were taken.

### 2.4 Data analysis

To facilitate a process of data familiarisation, interviews conducted in English were transcribed by the primary author (MH) and interviews using Bangla were transcribed by RB. The transcription technique followed an intelligent verbatim approach, using a word-for-word account and noting additional non-verbal cues (e.g., long pauses, laughter) where deemed significant [45]. Transcripts were then entered into the qualitative data analysis tool QRS International NVivo (ver. 12.6.0.959) for analysis. Paper notes and data familiarisation maps were used in addition to software to maximise data interaction and develop interpretative insights [46].

Interview data was coded reflexively using a blended coding approach [47]—a combination of deductive and inductive coding. Initially, data were coded deductively using broad categories from agency theory i.e., information asymmetry, opportunism and incentive structures/agency cost. New code categories were added inductively during analysis. Codes were discussed with a second researcher (PA) during data analysis to ensure rigor in the analysis. Manifest content analysis, a type of qualitative analysis used to provide a structured approach towards descriptive accounts of interview data [48, 49] was used to describe contract broiler production and strategic decisions leading to antibiotic use. Reflexive thematic analysis, following guidelines set out by Braun and Clarke [50, 51] was used to further explore the experiences of broiler stakeholders using an agency theory lens. Themes were generated using principles described by Braun and Clarke [51] and Connelly and Peltzer [52] and created to explain stakeholder decisions. In our analysis, we take a critical realist ontological and contextualist epistemological position. This theoretical positioning was adopted to recognise how we sought to uncover ‘a truth’ regarding antibiotic use, but how access to this version of reality was dependent on participants experiences and interactions between the local context and research team. Furthermore, this project formed a central part of the primary authors’ Ph.D. thesis, a thesis with a focus on antibiotic use and resistance. Thus, ideas about the importance of antibiotic use, resistance, and stewardship, were deeply entangled in the underlying framing of the project. This framing is further entrenched through the institutional and political environments of the core research team–antibiotic resistance policy being on the RVC’s and WBUASF’s research and UK’s and India’s political agendas.

### 2.5 Ethics

Pilot interviews were conducted under the OASIS project ethical approvals granted by the London School of Hygiene and Tropical Medicine’s Observational Research Ethics Committee (ref. LSHTM-IECHR-01-2019) and the Indian Institute of Liver and Digestive Service’s Institutional Ethics Committee for Human Research (ref. ILDS-IECHR-01-2019). Ethical approval for round one and round two of data collection was granted by the Royal Veterinary College’s Social Science Research Ethical Review Board (ref. URN SR2021-0095 & URN SR2021-0196 respectively). Additional information regarding the ethical, cultural, and scientific considerations specific to inclusivity in global research is included in the Supporting Information (S5 File).

## 3. Results

### 3.1 Production systems of study participants

Broiler production in our study participants primarily consisted of commercial broilers (e.g., Ven Cobb 400) raised under formal production marketing contracts. These contract farmers (n = 17) were all male, aged 23 to 63 years (median 46.5), with varying educational backgrounds (from illiterate to graduate level) and experience in poultry farming (from 6 months to 30 years). The four remaining farmers in the study raised other types of broiler breeds outside of contract arrangements, i.e., farmers bought chicks in cash and arranged bird sales themselves.

Many participants talked about how broiler farming was an opportunity to move away from poorly profitable crop production, for example:

*“When I did agriculture, I earned less money. So, for better income, I started [broiler farming].”* 220303_1507 –Broiler farmer

Farmers worked with a range of different companies operating in the West Bengal; Venky’s, Suguna, Premium, Rupa, Hitek, Shalimar, IB, Basu, and BK Roy and most had worked with more than one company. None of the farmers, however, were able to provide copies of their contracts. Consistent with the literature on contract broiler farming [2], these companies supplied farmers with production inputs (day-old-chicks, feed, and medicines) and farmers supplied land, labour, and bedding material, and raised birds for around 35 to 42 days. Several chick and feed dealers described how farmers in the state had previously bought chicks and feed from them, but these arrangements had become less frequent over the last few years, fueled by crises such as seasonal cyclones and the COVID-19 pandemic:

*“I had 30,000 birds [a month] business and it was ok. Due to COVID I [became] detached from broiler [production]. I am also a farmer, I also do broiler at my home, but production is off at this moment. […] COVID destroyed everything. […] Ten years ago, 97,000 chicks a month were placed.”* 220227_1238 Feed and chick dealer

To compensate for their loss in sales of commercial broilers, some feed and chick dealers were now trading in non-imported broilers hybrids and reported placing around 3000–4000 chicks a month across 10 to 15 farms.

Farmers talked about the local permissions they needed to begin production. Before building sheds, farmers had to apply for a ‘no objection certificate’ from the local authority (the panchayat office). This certificate demonstrated that the farmer’s neighbours did not object to the potential noise and smell of broilers. However, one farmer talked about how after receiving his permission and building a shed, the permission was revoked, and he had to re-build in another location:

*“I took permission from panchayat but later they told [me] that you have to take the permission again, so I was bound to destroy the shed. Then again, I built a new shed and made documents also. […] It’s party politics. [There was a] case against me. So many problems. Within thirty days I had to shift.”* 220303_1507 Broiler farmer

Broiler sheds generally held between 1000 and 2000 birds, with reports of a few farms housing up to 10,000 birds. The level of financial investment for broiler farmers varied between 60,000 Indian rupees (INR) (approx. 780 USD) to 475,000 INR (6175 USD). Most farmers reported using their savings from agriculture, or in some cases, borrowing money from relatives and friends, to start their enterprises. Several farmers described difficulties in accessing bank loans to start broiler production which they ascribed to a bias against broiler farming:

*“I tried for a loan, but bankers told [me] that ‘we have no loan for poultry farming’.”* 220224_1149 –Broiler farmer

One farmer described how he used his reputation as a successful fishery businessman, to access a loan from a bank, which he then used to invest in broiler farming. Those farmers who could not access large amounts of financial capital were forced to rely heavily on using local materials, such as bamboo, to construct poultry sheds:

*“It did not require much money. Bamboo pillar from my garden. Part of the money was taken from agriculture savings.”* 220224_1559 –Broiler farmer

**Fig 1:**
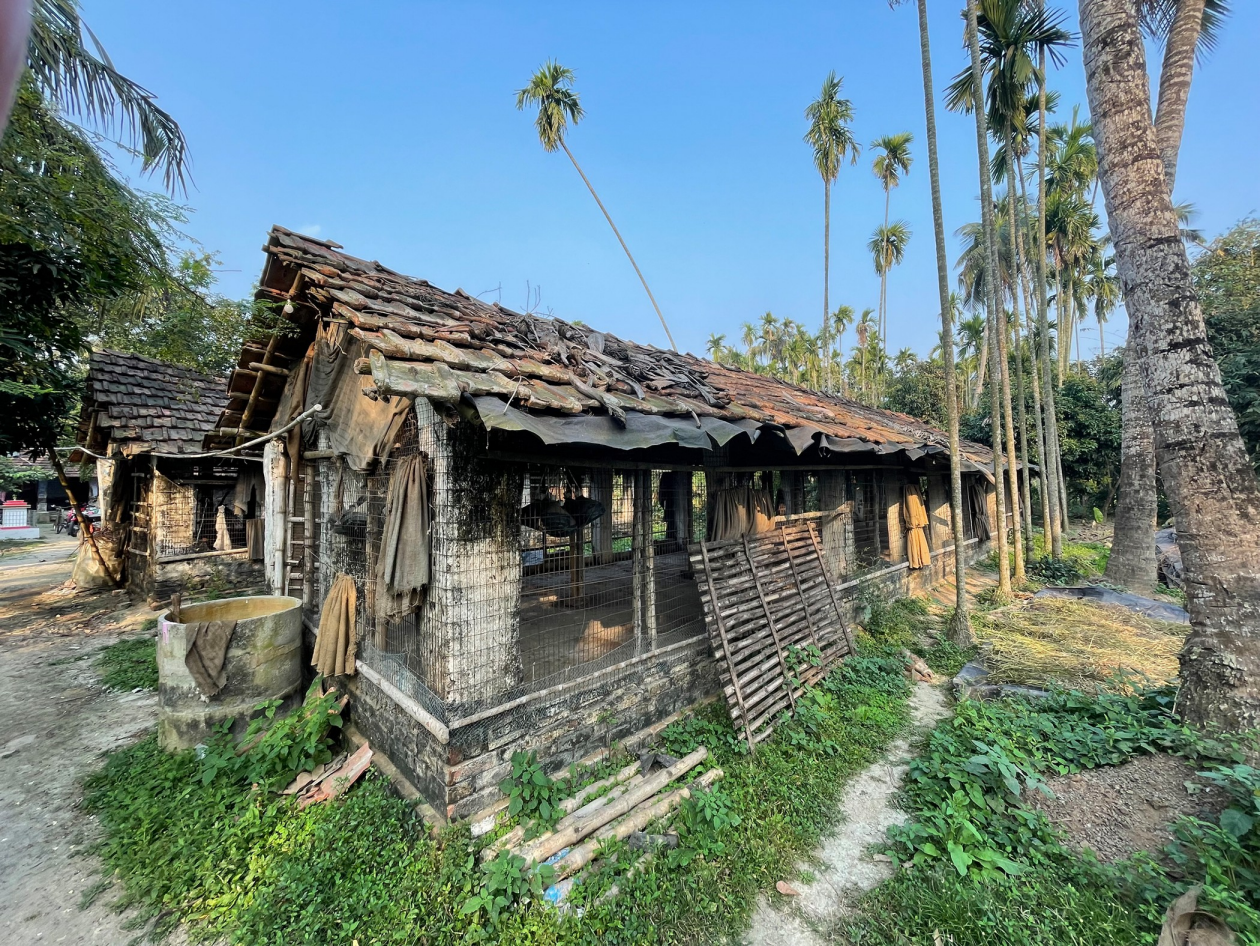
Photograph of typical ‘open housing’ for broiler chickens.

Thus, the quality of poultry housing, and associated farm biosecurity, varied between farmers with only some being able to construct sheds using more durable materials such as concrete and asbestos (While asbestos as a building material has been banned by many countries due to the associated risk of mesothelioma (UK ban 1999) it continues to be used as a building material in India which engages in a large import industry). Typically, these ‘open’ sheds had limited environmental control (Fig 1) and were prone to pests such as rats which could burrow through earth floors.

The lack of environmental control was a concern for many contract company stakeholders and farmers, particularly for fluctuating temperatures and extreme heat and humidity during the summer, all creating production challenges:

*“Temperature fluctuations are there, there is no control over humidity. In summer it can go up to 42 to 45 Celsius, and in winter it can be 5,6,7,8 Celsius. This is one of the factors which can impact the performance of the birds and prevent their full genetic potential.* 210427–1609 Nutritionist, poultry consultant

Even those companies which have their own semi-environmentally controlled housing were only able to partially manage the production environment. Here, ambient temperature could be moderated using fan systems to pump air through housing, but humidity was not controlled. These semi-environmentally controlled sheds were reported to cost around 5,000,000 INR (65,000 USD) to construct and could house around 11,500 birds. One contract company general manager believed there to be around only 50 of these sheds in West Bengal out of an estimated 16,000 broiler farmers.

Farmers reported having three to six cycles per year and most reported getting better profits in the winter season, as high ambient temperatures in the summer caused higher poultry mortality. Maximum revenue for each production cycle was reported as between 6,600 INR (86 USD) to 26,000 INR (338 USD) per cycle of 1000 birds, though some reported making a loss during a production cycle, for example:

*“I got profit in the first three to four lots but after bird flu disease came I got a loss. Again, I did three to four lots but no profit.”* 220226_1330 –Broiler farmer

This variation in profitability was also reflected by a branch manager talking about his company:

*“Our production was so good that only 53 farms got nil payment. More than 1000 farms got super payment. Other companies gave nil payment to 30% of their contracted farms. So, by this, new farmers come to us, and we explain our system.”* 220226_1414, contract company branch manager

At the end of the production cycle contract companies would arrange to collect birds for sale, a process known as ‘lifting’. After lifting, farmers usually keep their sheds empty for around two weeks to clean the sheds and allow them to sit empty between batches of birds. Farmers were paid a growing charge for each kilogram of broiler produced. Though farmers did not have to pay for chicks, feed, and medicines, they did have to pay for electricity and rice paddy husk, used as bedding/litter material in the sheds, which cost around 5,500 INR (72 USD) per cycle per shed. Therefore, in situations where major production losses occurred, such as due to disease outbreaks, then farmers risked losing money during production as they did not receive compensation from poultry companies nor the government.

Four interviewed farmers (one of which was female) were engaged in independent (non-contract) production of other chickens. These chickens included native birds known as ‘deshi’, or Rhode Island Red hybrids known locally as Sonali, Boran, or Kuroilers. Hybrid birds took around two to three months to reach slaughter weight. These independent farmers raised a smaller number of birds (200 to 1500) and engaged in two or three production cycles per year. One of these farmers talked about how their lack of financial capital and concerns over not being able to make a profit as reasons for not engaging with contract broiler farming:

*“[Contract farming] requires a lot of money, I have to build sheds. Do you know the company never gets a loss? […] They will always make their profit and after that if something is left then only then they give 5000 to 10,000 rupees [65 to 130 USD]. Otherwise, it will be a zero balance. They never look at the electric meter or shed rent. So, I am not interested in company business.”* 220302_1309 Rhode Island Red producer

One woman raised Sonali birds for both meat and egg purposes. She obtained day-old chicks from both a local dealer and by hatching some of her own eggs using a small incubator that could hold around 100 eggs.

#### 3.1.1 Antibiotic use during broiler production

During the production of imported commercial broilers, antibiotics were used to control and prevent disease and protect against associated losses. Many of the contract company stakeholders talked about how antibiotics were given prophylactically to day-old chicks within the first three days to protect against *Mycoplasma* infections and naval ill caused by *Escherichia (E*.*) coli* bacteria. During the production cycle, antibiotics would be given therapeutically if there was evidence of diseases such as chronic respiratory disease caused by *Mycoplasma* species, colibacillosis caused by *E*. *coli*, or during outbreaks of viral disease—e.g., Gumboro (infectious bursal disease), Newcastle disease and avian influenza–to prevent secondary bacterial infections. Many stakeholders held the perception that water used on broiler farms was a major cause of *E*. *coli* infection in birds:

*“If it is found that an area is having a high risk of enteric infection because of problems like water quality, then a decision is made that antibiotics are [used]. In other areas where water quality is good when the weather is good, then the decision is taken at the top level that antibiotics will not be used.”* 210427–1609 Nutritionist, poultry consultant

Antibiotic growth promoters (AGPs) were reportedly still being used to improve productivity and protect against production losses. Typically, AGPs were added to feed by manufacturers and may be used during the entire production cycle. Though colistin (a polypeptide antibiotic) has been banned since 2019, some stakeholders had concerns it was being used illegally by a small number of producers. Except for chlortetracycline, a tetracycline, antibiotics used for growth promotion belonged to classes not being used for therapeutic and prophylactic use. Several stakeholders talked about the classes of antibiotics being used for growth promotion as being ‘gut acting’ antibiotics, i.e., considered not to have systemic activity:

*“In the case of broiler chickens–these antibiotics are not absorbed across the lumen of the intestine. For example, enramycin, it does not get absorbed. So, if these antibiotics are in the feed the chance of getting them in the meat is very less. […] The regulatory authority should consider whether it is being absorbed into the system, if it is not absorbed in the intestine, then ok they can be given permission.”* 210427–1609 Nutritionist, poultry consultant

A summary of those antibiotics mentioned during interviews with contract company stakeholders and farmers is provided in Table 3.

**Table 3:** Classes of antibiotics reportedly being used during broiler production.

Antibiotic class	Therapeutic and prophylactic use	Conditions being used for	Growth promotion	Considered critically important for human health [53]
Fluoroquinolones	Enrofloxacin, levofloxacin, ciprofloxacin	Respiratory disease/pneumonia		Yes
Macrolides	Tylosin, tilmicosin	*Mycoplasmosis*		Yes
Tetracyclines	Doxycycline	*E*. *coli infections*	Chlortetracycline	No
Aminoglycosides	Gentamicin, amikacin, neomycin	*E*. *coli infections*		Yes
Polypeptides			Bacitracin dimethyl disalicylate, enramycin),	No
Streptomycins			Virginamycin	No
Glycolipids			Flavomycin	No
Lincosamides			Lincomycin	No

When asked about medications and antibiotics, most of the contract broiler farmers we spoke to reported how these decisions were taken by the company supervisors and veterinarians. One contract farmer, who also had experience working with a fishery cooperative, talked about his desire to see broiler production move away from routine antibiotic use:

*“I want more improvement, more developed farm. And if it is possible to leave the antibiotics and use probiotics. […] We have already stopped using of antibiotic in fish and using only probiotics.” 220225_1643 –Broiler farmer*

Some broiler farmers were able to name antibiotics used during production, such as neodox (neomycin and doxycycline produced by Cargill) and Enrocin (enrofloxacin 10% produced by Zoetis), but others did not know what type of medicines were used and defaulted responsibility to the company. Similarly, the non-contract farmers we spoke to reported relying on poultry shops or local healthcare providers to prescribe medicines.

### 3.2 Contract broiler farming through an agency theory lens

Three themes were generated from the interview data to provide insight into key strategic decisions being taken during contract broiler production. Here, the themes ‘Production occurs in challenging environments which requires risk mitigation’, ‘Concerns over breeding practices leads to antibiotic use’, and ‘Farmers’ uncertainty over input quality drives contract mobility’ are presented along with their framework elements (information asymmetry, opportunism, and agency cost).

#### 3.2.1 Theme 1: Production occurs in challenging environments which requires risk mitigation

**Fig 2:**
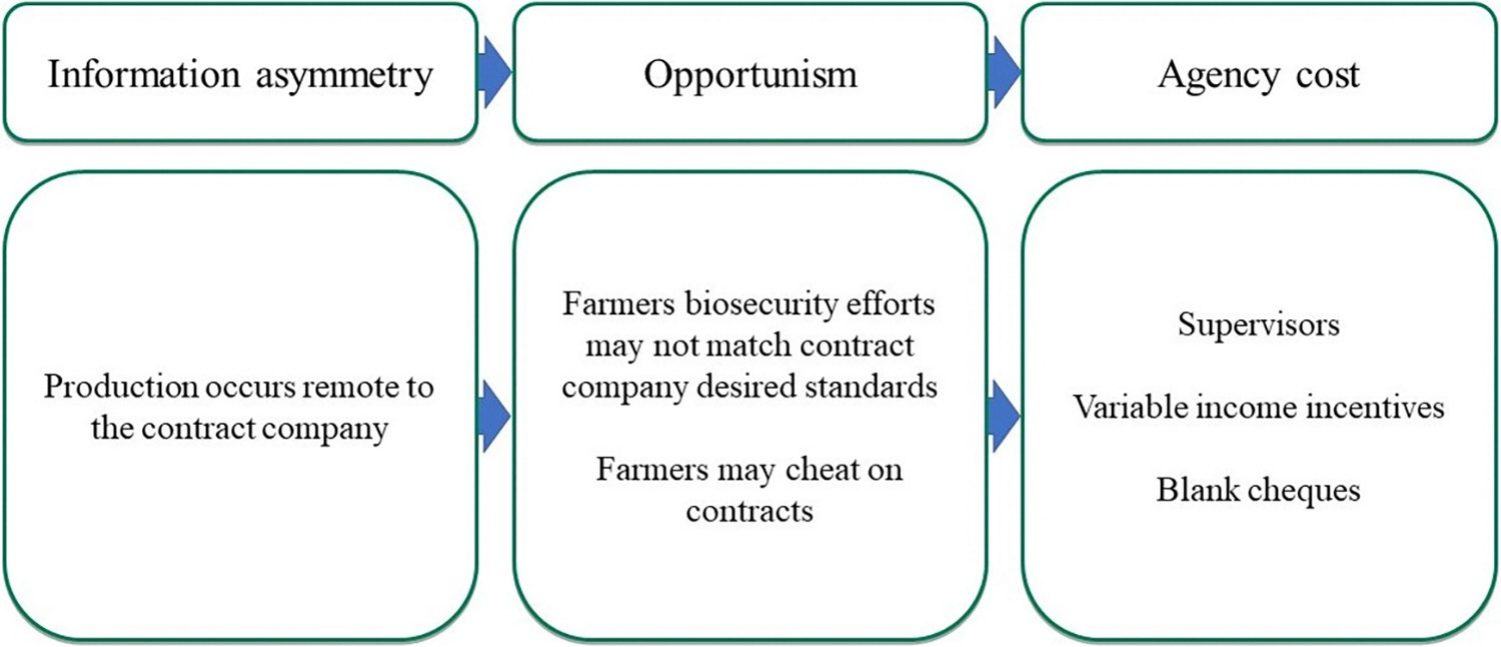
Framework elements for theme 1.

In this theme, we explore how a decentralized production system creates challenges for contract companies which must be mitigated through various methods (Fig 2).

As broiler production is decentralized, i.e., it occurs through numerous farmers located remotely to the company, information asymmetry can occur regarding production practices. Here, contract companies are not privy to all the production decisions taken on the farm raising concerns about the level of biosecurity deployed by farmers. Some contract company stakeholders talked about how biosecurity effort can vary between farmers, for example:

*“[Farmers] compensate for their lapse in biosecurity; people coming from outside, farmers entering the farm from markets. They are using more antibiotics, and more medicines, to compensate for this lapse. […] If one farmer is doing strict biosecurity and others are not, then he also suffers. ‘If I do and others are not doing, then what is the point?’”* 210326_1911 Veterinarian, poultry consultant

Some farmers were reported to engage in cheating behaviour, e.g., selling feed and birds outside of their contract. Here, farmers may choose to sell feed or birds into the private market outside of the contract arrangement to make additional money:

*“Farmers in this culture feel they do not get enough profit, so they start misbehaving; he will start thieving the chickens. They will eat some chickens. […] He will sell it at the nearby farm gate sometimes–so at that time he will get listed as a black-labeled farmer–sometimes the agreement is void.”* 210330_2026 Veterinarian, academic

Consequently, contract companies expend agency costs to reduce this potential opportunism. Companies employ supervisors to monitor production. Within these supervisor systems, employees of contract companies who have received training in broiler production visit farms daily or every few days. Branch managers described how they managed between seven and 15 supervisors per branch, each of which looked after 24 to 30 farms. These supervisors collect information on broiler production; feed consumption, medicine usage, and broiler mortality which is relayed back to the company and used to guide production decisions. In response to potential cheating behaviour, companies collect several blank cheques at the start of a contract arrangement. The purpose of these cheques was to act as a disincentive for farmers to cheat on their contracts, i.e., should cheating occur then companies could cash in these cheques at banks to recoup losses.

**Fig 3:**
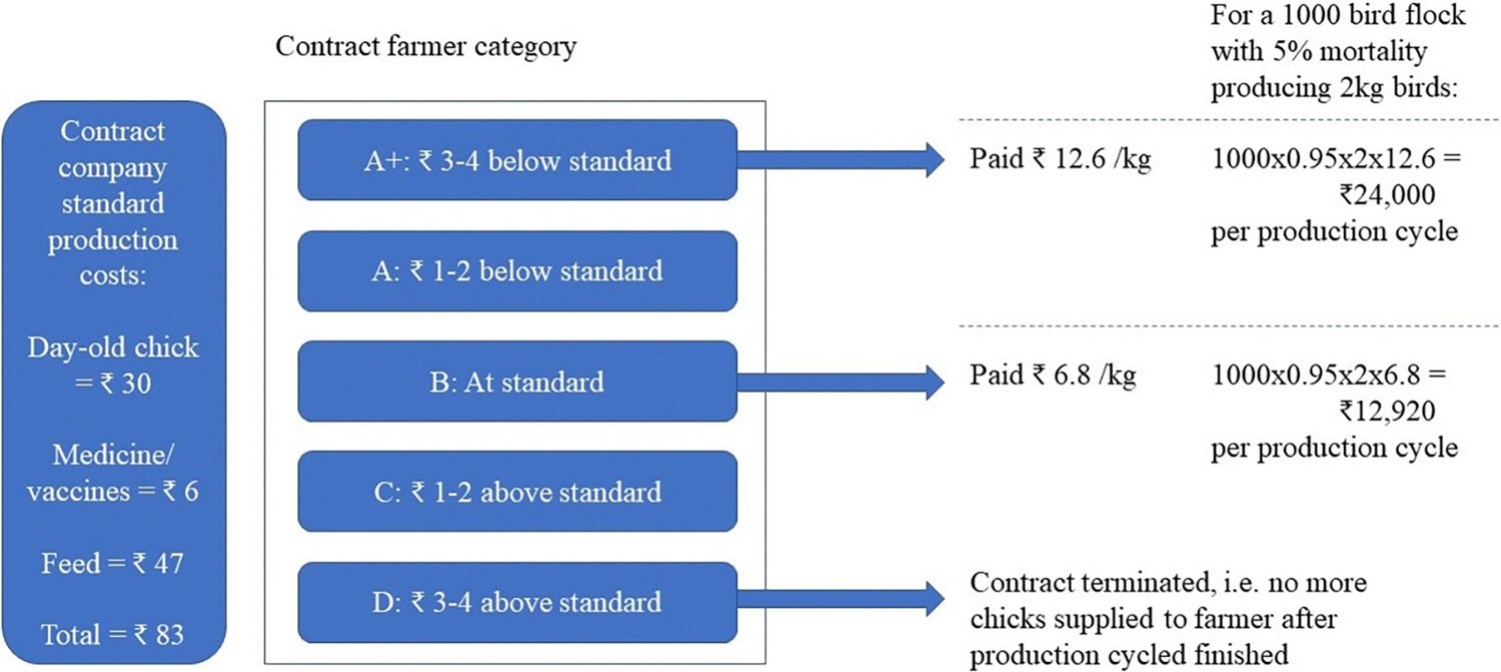
Variable income incentive structure used by one contract company (as described by 220324_2016 poultry consultant). Costs in Indian rupees (1 INR ~ 0.013 USD at time of fieldwork).

The growing charge received by the farmer was dependent on how the total production cost (chicks, feed, medicines) compared to the company standard. A poultry consultant working for one of the large contractors described this process for their company (Fig 3). Here, farmers working for this company were graded depending on how their production cost compared to the company standard. Farmers who were able to produce chicks for the same as the company standard would be paid 7 INR (0.09 USD) per kilogram of broiler produced, equating to around 13,000 INR (169 USD) per thousand birds produced. The most efficient farmers were paid a growing charge almost twice the company standard, whilst those producing too far above the standard were at risk of having their contracts terminated.

The collection of information by supervisors was used to optimize decisions to mitigate the risk of producing broilers in challenging environments. Here, antibiotic use decisions were taken by companies, with probiotics being used in well-performing farms and prophylactic antibiotics in the others:

*“Right now, if you visit one hundred farms, twenty will use probiotics so eighty will use antibiotics. […] We always insist that if you run your farm properly, clean your farm, use disinfectant, and then go for twenty days’ rest. If the previous lot was excellent there is no need to go with antibiotics, use probiotics.”* 220321_1220—Contract company general manager

However, while many stakeholders reported increasing use of antibiotic alternatives in the sector, these products were reported to have limitations. Firstly, they are costlier than antibiotics, and secondly, they were not considered to be as effective at supporting production in particularly challenging settings. Consequently, some stakeholders consider the removal of antibiotics to be problematic:

*“Since the birds are always challenged with enteric concerns, if we withdraw antibiotics altogether there will be some problems.”* 210427_1609—Nutritionist poultry consultant

An alternative strategy for contract companies to deal with production in challenging environments is to move production in-house, i.e., removing/reducing the asymmetry of information regarding biosecurity control. One stakeholder talked about how they had taken this move to enter the market of antibiotic-free production:

*“For our processing plant, we have taken the decision that we go for antibiotic free. For that reason, we have constructed our [own] few farms, we can maintain our chick quality, we can maintain our biosecurity. Until now we are successful.”* 220321_1220—Contract company general manager

This type of vertically integrated production, however, appears to be in its infancy in West Bengal. As previously described, these systems are expensive to construct and currently only provide partial environmental control.

#### 3.2.2 Theme 2: Concerns over breeding practices lead to antibiotic use

**Fig 4:**
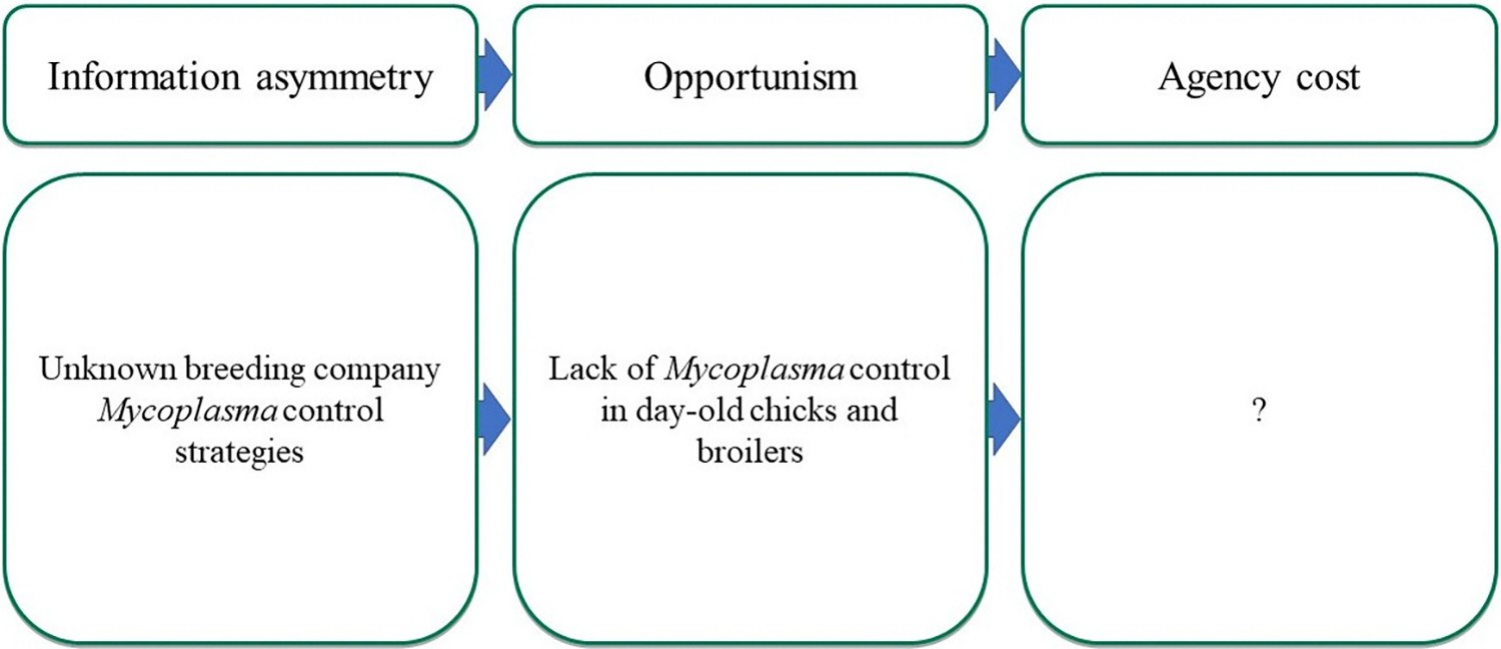
Framework elements for theme 2.

In this theme, we explore the concern shared by many contract company stakeholders about the level of *Mycoplasma* control being invested in by broiler breeding companies and how this results in the use of antibiotics belonging to classes deemed critically important for human health (Fig 4).

Many contract company stakeholders reported concerns about the unknown *Mycoplasma* status of day-old chicks being bought from breeding companies. This represents an example of private information between these two stakeholder groups. Here, some contract company stakeholders had concerns that breeding companies were inadequately controlling *Mycoplasma* infections in their stock as control strategies were difficult to implement:

*“In [India] mycoplasma-free chicks are not available. Mycoplasma with breeder is not being taken seriously- vaccines are available but not available all the time and [the] chicks are carrying maternally derived Mycoplasma from vertical transmission. The [breeding companies] are doing business, so they are easy.”* 210326–1911 Veterinarian, poultry consultant

It was the perception of some contract company stakeholders that adequate *Mycoplasma* control was not occurring in breeding stock due to high associated costs:

*“Hatchery people, unless they have their integrators, will not take care. Mycoplasma prevention for breeders is a very costly affair. When there is a change in the market, particularly chick sales [fall], they compromise Mycoplasma control. Most of the breeders are still on [antibiotic] control programs and not vaccinations. So, most of the supply of chicks have Mycoplasma in [some] level.”* 210525–1842, Veterinarian, academic

Consequently, day-old chicks arriving on farms were presumed to be infected with *Mycoplasma*, and antibiotics were used to mitigate this risk:

*“See our chicks are not Mycoplasma free so there will be problems. Antibiotics [are given] on the first day, that is the common approach from veterinarians. […] Without antibiotics there are some issues, performance maybe a little bit poor.”* 210326–1911 Veterinarian, poultry consultant

It was reported by several contract company stakeholders that tylosin, a macrolide antibiotic, was routinely used at the start of broiler production to compensate for the lack of *Mycoplasma* control, and enrofloxacin, a fluoroquinolone antibiotic, used at later stages of production to treat chronic respiratory diseases associated with *Mycoplasma* infections. While the use of antibiotics is a cost associated with this potential deviation in the behaviour of breeding companies concerning producer’s needs, interviews did not identify decisions consistent with agency cost where companies can incentivise breeders to act differently.

#### 3.2.3 Theme 3: Farmers’ uncertainty over input quality drives contract mobility

**Fig 5:**
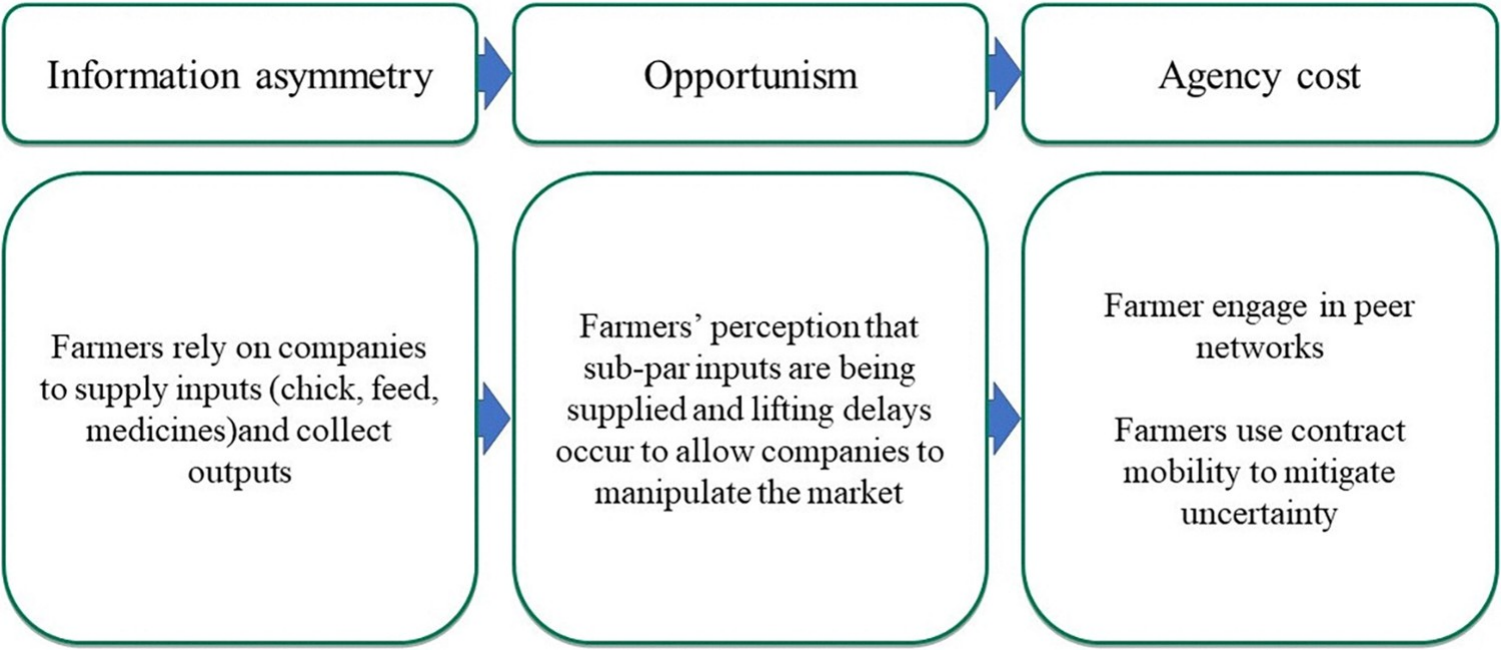
Framework elements for theme 3.

In this final theme, we explore the contract relationship from a different perspective. The agency lens is flipped to consider how broiler farmers seek income opportunities from contract companies. Here, we consider the type of information broiler farmers feel they do not have adequate access to and the potential consequences (Fig 5).

Many farmers talked about their concerns with the quality of inputs (chicks, feed, and medicines) being supplied to them. Here, poor quality inputs are blamed, at least partially, for poor profits, for example:

*“Good quality chicks are not supplied. If the company changes this, then it will be better for us.”* 220224_1327 –Broiler farmer

*“Broken rice was mixed with the feed*. *They [contract company] were sending finisher [feed] instead of a starter*.*”* 220225_1643 –Broiler farmer

*“Sometimes I also buy medicine from the market*. *[…] [Contract] company’s medicine is not so efficient […] [They] give low-quality medicine*.*”* 220224_1149 –Broiler farmer

This concern over input quality, with farmers having to accept what they are given by contract companies, represents a perceived asymmetry of information concerning input acquisition.

Several farmers also talked about their concern over delays in lifting birds from their farms at the end of production cycles. This was considered problematic due to increasing mortality and increases in feed conversion ratios of birds towards the end of the production cycle:

*“If the [contract company] gets delayed picking the birds for four to five days, then some birds die and FCR [feed conversion ratio] goes [up] and the result is borne by the farmers only. My profit goes down and bonus money we do not get at that time.”* 220226_1659 –Broiler farmer

As farmers’ revenue is calculated using a growing charge which is proportional to input expenses farmers bear the cost of lifting delays and consequences of poor input quality.

In response to the previously described information asymmetry, farmers can be considered to expend agency cost in two ways. Firstly, they can expend social capital and engage in peer networks to increase their knowledge of other companies’ practices. Secondly, they can terminate their contract and move to another company, thus providing a disincentive for company practices which are undesirable for farmers.

When describing how they made production decisions, many broiler farmers talked about their peer interactions, which were often with people in their local area. Several people talked about how they made the initial decision to engage in broiler farming by talking with friends, family, and neighbours who had worked in the sector:

*“I noticed when I used to roam here and there by bike and bus. I [saw] so many farms and asked the farmers about the shed, profit and management. One person helped me about the height of the shed and pillar and so I started, and I found a labourer and guided him how to build the shed.”* 220224_1149 –Broiler farmer

These types of interactions were also useful in helping farmers make decisions regarding their ongoing contract arrangements. When faced with poor profits from production cycles, information gathered from people working with other companies would help inform future contract decisions:

*“So many farms in my village, someone is with [the company] Basu, some with Hitek, some are with Suguna. Farmers advise to move [to those] who are getting profit.”* 220226_1330 –Broiler farmer

Only a few farmers talked about using data gathered from more remote sources, such as advertising or the radio to help inform their decisions:

*“I used to listen to the radio and from [there] I knew that broiler [farming] is a profitable business. Suguna is a good company. […] The radio and TV broadcast chicken prices of Suguna. That way I knew that Suguna is a big company.”* 220224_1559 –Broiler farmer

These peer-to-peer networks appear to be mostly informal; farmers said they did not belong to groups or organisations of broiler farmers, though one farmer did talk about attending a meeting organised by a contract company for their contracted farmers:

*“Company calls a meeting. […] Suppose twenty people are there. One meeting is called. […] Once in a year, once in six months. No fixed time.”* 220303_1355 –Broiler farmer

While some broiler farmers reported working only with a single company, most had worked with multiple. Farmers described moving from one company to another to seek a better outcome for their business. As previously reported, farmers described how profits varied from one production cycle to the next, and in some instances were zero. Consequently, farmers experiencing successive poor profits looked to move to another company, if possible, to improve their situation, and this behaviour may be adopted by others in the local area:

*“After doing the business for one year, if I do not make a good profit then I will change [company] and at the same time if somebody is giving more profit then I will change. [It] is very common here that all farmers change at the same time. Maximum of thirty to thirty-five farmers are here. All will change the company at the same time.”* 220303_1355 –Broiler farmer

Some farmers reported only having the choice of two companies to work with while others worked in areas where several companies operated. One farmer talked about how they could even return to a previous company when they so wished, suggesting this tactic did not always damage the relationship between the two parties:

*“When we face problems with doctors, problems with feed and sometimes problems with chicks then we move towards another company. And we can also get back to our old company. This is our system. I tell them “Dear, I am changing your company and when I will get interested then I will join again.”* 220303_1355 –Broiler farmer

Most farmers described the process of moving between companies to be a relatively easy task. Thus, the act of contract mobility appears to be a key strategy in farmers’ ability to navigate contract farming, though one limited by the availability of alternative options.

### 3.3 Key strategic decisions during broiler production affecting antibiotic use

**Fig 6:**
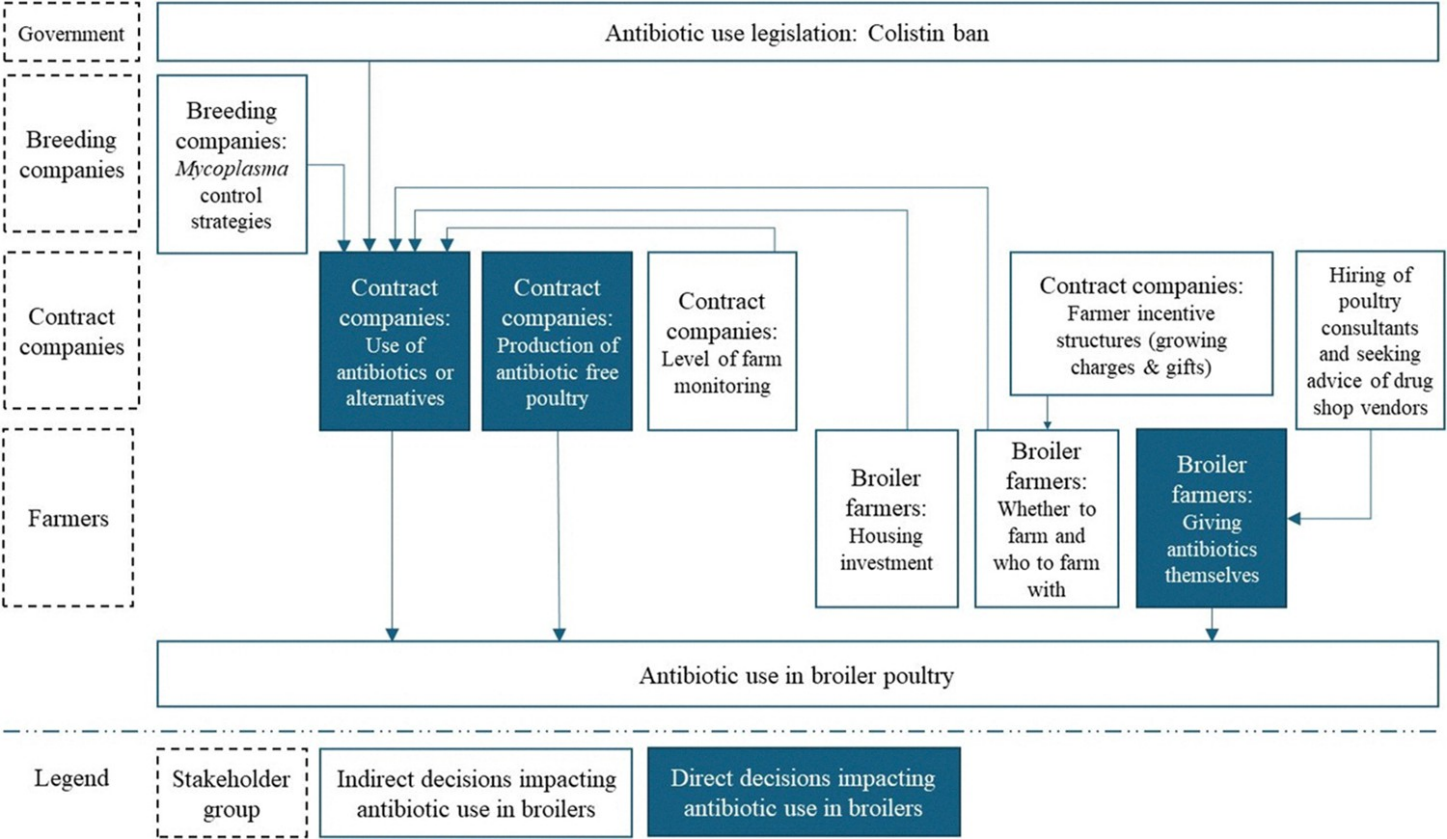
Interactions between strategic decisions affecting antibiotic use in contract broiler farming: Decisions having a direct impact (blue boxes) and those having an indirect impact (white boxes).

In this final section we summarise the various strategic decisions which impact antibiotic use during broiler production (Fig 6), several of which have been described in the previous sections. Here, decisions are stratified by the level of stakeholders in the production network and classified as having either a direct or indirect effect on antibiotic use.

#### 3.3.1 Government and the poultry sector

While antibiotic use appears to remain common during broiler production, a key strategic decision that took place in 2019 was the nationwide ban on the use of colistin, a polypeptide antibiotic, in livestock. Before this ban, colistin had been used frequently in poultry as it was water soluble and an effective treatment for common bacterial diseases. While the government has yet to further restrict access to antibiotics in the poultry sector, several contract company stakeholders talked about how their decisions to invest in antibiotic alternative research was due to a concern that further legislation would be introduced.

#### 3.3.2 Contract companies and broiler farmers

Antibiotic use practices appear to vary between contract companies, with some reportedly using more antibiotic alternatives than others. Therefore, farmers’ decisions on whether to engage in contract broiler farming and which company to work with will indirectly affect antibiotic use. Farmers’ decisions about which company to work with are dependent on company decisions around the incentive structures (growing charge arrangements and informal incentives) offered to farmers.

Farmers’ decisions around housing investment impact contract company decisions around antibiotic use, with some companies only using antibiotic alternatives in those farms considered to have better biosecurity practices. However, farmers’ housing investment decisions are also connected to their ability to access credit, with decisions regarding credit access being taken by both contract companies and banking institutions.

While most farmers talked of how they adhered to the stipulations of their contract and followed the advice of supervisors regarding antibiotic use, some reported treating broilers on their own, taking advice from poultry consultants or drug shop vendors i.e., making a strategic decision to act outside of contract.

## 4. Discussion and considerations for antibiotic stewardship

Using a theoretical framework provided by agency theory, we examined the relationships which occur during contract broiler production in West Bengal, India, where small and medium enterprises dominate. Due to the inherent nature of contract livestock relationships, information asymmetry around production practices exists and antibiotic use remains a dominant risk mitigation strategy for contracting companies, though concerted efforts to move away from this are occurring. Should India’s poultry move towards large scale production where companies are in direct control of operations, as has occurred in many high-income countries, then the magnitude of information asymmetry would likely reduce. This type of agrarian change, however, could jeopardize the livelihoods of the 50 million people to which the sector provides direct and indirect employment [54]. Encouragingly, the Indian government has recently produced recommendations and guidelines to protect the interests of small and medium size broiler enterprises operating in the sector [44, 55].

While participants indicated that contract broiler farming was an opportunity for farmers to generate income, this opportunity contains uncertainty. The broiler farmers in this study exist in an uncertain production environment where they sometimes experience negligible profits and other times losses. This type of uncertainty during contract broiler production in India has been reported by others [20, 56]. This contrasts with Sasidhar and Suvedi’s 2014 study [2] which describes contract broiler farming in Karnataka, Telangana, and Andhra Pradesh as giving “a lower but assured and almost fixed return” (p. 38) compared to non-contract farming. At the time of their study, the authors reported that 37% of broiler production is under contract, which is reflected in their comment that farmers can make active choices about whether to produce broilers independently or under contract. Over the last decade, however, contract farming has come to dominate the broiler sector in India, with recent estimates indicating that around three-quarters of broiler production occurs under contract [45, 57]. Independent broiler production does not now appear to be a viable option for most producers in our study area. These farmers lack the financial capital, or access to loans, to ‘go it alone’ and farm independently or under informal output-marketing contracts. Further research would be necessary to ascertain whether our findings are true for other Indian states, and whether the dominance of contracting has weakened the bargaining power of farmers in their ability to generate income from broiler farming.

Broiler farmer’s concerns about poor input quality needs further investigation. These perceptions may reflect an underlying reality, where poor quality inputs are to blame for poor outputs. Or they may represent a fallacy, where input quality is adequate and unsatisfactory outcomes are a result of other factors, such as high burdens of disease. In their study of contract broiler farming in Tamil Nadu, Thamizhselvi and Rao [56] describe how contracts are one-sided as they stipulate output standards but not input quality standards. Here, the authors report concerns with low day-old chick weight as a production issue for farmers. If these practices occur, then farmers need ways of increasing their bargaining power in contract relationships to avoid one-sided arrangements. Future work could investigate whether the new broiler contract guidelines [55], which stipulate that “provided inputs will be as per the applicable norms, standards, and regulations” (p.3) will change this and empower farmers. Those contract companies supplying quality inputs, however, may benefit from engagement with the theory of testaments [27]. Here, companies would build trust through communication channels to assuage farmers’ concerns about low quality, thus helping to address this asymmetry of information from the point of view of the farmers.

Situating broiler farmers’ perceptions of contract farming within an agency theory framework, we observed how social capital is used to navigate contracts. Social capital–the economic value of personal relationships–is considered to contribute to farmers’ self-efficacy and a belief in their ability to perform [58]. For the farmers in our study, social capital appears restricted to local networks as broiler farmers described not being part of formal associations. Thus, their source of information appears limited to their peers or contract companies. On this note we may reflect on Mitnick’s [24] original contribution to agency theory–the institutional theory of agency. Here, Mitnick focused on the institutions which form in response to imperfections of agency relationships. For the West Bengal broiler farmers in our study, imperfections in the contract relationship (production in decentralized and challenging environments and unreliable farmer income) have created local knowledge sharing social networks. These networks, however, have not led to the creation of formal institutions and associations for broiler farmers. In their study of poultry farming in Kenya, Kithendu [59], describes how the formation of co-operatives allowed members to build social capital and access knowledge and technology. Interestingly, during our study, some participants talked about their involvement with fishery co-operatives in the region, and schemes supported by government livestock extension officers, but parallels did not exist in the poultry sector. Establishing a functioning broiler cooperative could therefore be one route to provide legitimacy to the informal social peer networks and act as a conduit of knowledge and technology sharing. This may also help improve loan access–identified as a barrier to infrastructural change—as some farmers had explained they were able to access bank loans via their association with fishery cooperatives. Given the uncertain profitability of broiler farming to small and medium enterprise farmers reported here, it seems unsurprising that banks consider this to be a high-risk business and are unwilling to provide farmers with loans. Further work, however, would be necessary to understand the decisions being made by financial institutions regarding access to capital flow for broiler farmers. Microcredit schemes have been used in the Vietnamese poultry sector, allowing farmers opportunity to invest in biosecurity measures such as vaccination and restructuring of poultry housing [60]. This study did not, however, look at the subsequent impact on antibiotic use.

### 4.1 Implications for antibiotic stewardship

By questioning broiler stakeholders about their antibiotic decisions, we were able to get a sense of the level of use during production. Responses showed that antibiotic use appears to be common during broiler production in West Bengal. A variety of antibiotics were reportedly being used (listed in Table 3), several of which belonged to classes deemed critically important for human health [61, 62] which is concerning. Additional research is needed to understand several questions that this study has raised. This includes surveys to quantify antibiotic use, as well as work to understand how antibiotics are procured, and investigate the effectiveness and economics of current antibiotic use practices. Broiler production is a commercial enterprise, and it is therefore expected that farms adopt risk mitigation strategies to protect their interests [63]. Given the challenging production environment in which many Indian broilers are being raised, and the current varying confidence and increased cost of antibiotic alternatives, the status quo of ongoing antibiotic use seems unsurprising. In a study of antibiotic use in 288 broiler farms in Bangladesh, Chowdhury et al., found that 98% of farms had used at least one antibiotic during production and reported fluoroquinolone, macrolide, and colistin use in up to 22%, 8% and 3% of farms respectively [64].

Antibiotics are known to have ‘social power’ and their use as risk mitigation strategies is supported by other research [65–67]. Thus, stewardship efforts aiming to reduce antibiotic use in the broiler sector need to provide alternative ways of mitigating the innate production risk faced by producers. Here we discuss the relevance of our findings with respect to potential future antibiotic stewardship.

Infrastructural change, such as that offered through environmentally controlled housing or improved water quality may offer potential routes to reduce production risk. However, as previously discussed, contract broiler farmers face financial barriers to infrastructural change, and it seems likely that a move to environmentally controlled housing would need to come via the vertical integration of firms into production. While some firms we spoke to talked about this beginning to happen, even they acknowledged cost as a limiting factor for such a transition. Furthermore, despite semi-environmentally controlled housing being the predominant system in Pakistan [68], antibiotic use in poultry there remains high when compared to that of many high-income countries [69, 70] suggesting that simply changing housing is no sure fix to reduce antibiotic use. Alternatively, further research into antibiotic alternatives and methods of reducing their cost, may bolster confidence and help to reduce reliance on antibiotics as a main risk mitigation tool.

Contract company stakeholders’ concerns over inadequate *Mycoplasma* control in breeding stock and day-old chicks is of particular interest due to the ongoing use of macrolide and fluoroquinolone antibiotics used to treat this infection. While the antibiotic molecules tylosin and enrofloxacin are not used in human medicine they belong to classes of antibiotics deemed critically important for human health [53]. Avian mycoplasmas have been reported to be a cause of poultry respiratory disease worldwide resulting in significant economic losses for both breeder flocks and during broiler production [71]. A study of 60 broiler farms in Maharashtra found that 10% of flocks were positive for *Mycoplasma gallisepticum* and 20% positive for *Mycoplasma synoviae* [72]. Further work is needed to investigate whether the perception of inadequate *Mycoplasma* control in breeding flocks in West Bengal is true and if so, interact with breeding stakeholders to understand how control could be improved. For example, Aviagen, the producer of Ross broilers, has published protocols describing the testing schemes needed for *Mycoplasma* disease-free certification [73]. Currently, it seems stakeholders procuring day-old chicks have no mechanism to incentivise breeding companies to change production practices to *Mycoplasma* control, i.e., they cannot penalize suppliers by switching to companies with better *Mycoplasma* practices. This appears to be a structural issue that currently cannot be solved and requires further study to understand how this production dynamic can be improved as a route into antibiotic stewardship.

## 5. Limitations

Due to the dominance of contract farming in broiler production in West Bengal, agency theory can be considered a suitable social framework for studying strategic decisions leading to antibiotic use in the broiler sector. It is, however, a theory rooted in a neo-classical economic paradigm, particularly that of rational choice theory. Assumptions are taken that actors are rational individuals maximising profit-based utility, i.e., profits from the sale of broilers. Other factors traditionally considered ‘non-rational’, however, are neglected. Within the context of broiler farming, these may include job satisfaction, social standing within a community, or benefits gained from cooperative behaviour. The framework also considers power as a dyadic construct between two parties and is in the hands and disposal of agents. In the conventional use of the framework to examine contract broiler farming, these agents would be the farmers. However, whether farmers can take active choices around practices can be debated. Here, the decisions they take may be the result of surrounding structural forces leaving them with little opportunity for choice. While proponents of agency theory argue it provides a “unique insight into information systems, outcome uncertainty, incentive and risk” [74] (p. 57), critics argue it describes actors as opportunistic and lazy [75]. Nguyen [76] suggests that by being too focused on incentives and agency cost, agency theory does not consider the broader context in which social actors find themselves. Broiler production exists as part of a network of interactions that are influenced by a broad set of social, economic, political, and historical factors. Thus, further work to examine contract broiler farming in India could use alternative social frameworks. For example, stakeholder theory [77] could be used to examine the formation of relationships during commodity production, or critical political economics [78–81] used to analyse the political and socio-economic factors which have historically influenced the sector. This type of qualitative study could also seek to identify and analyse barriers to entry for those people currently not involved in poultry production and to further characterise the barriers for non-contract farmers entering into contracting.

The qualitative and explorative nature of our study represents one version of reality influenced by our choice of theoretical framing and the data generated from those we were able to interact with. As such, our findings require validation, as we have indicated through the suggestion of further work in the discussion. In addition to this, given the changes in the broiler sector over the last decade and a shift to contract production, research could be conducted to provide up to date comparisons between contract and non-contract systems, which was not the aim of our study. This type of comparative study could investigate the consequence of a move to contracting, such as differences in antibiotic use and profitability for farmers. However, as our study indicated, finding sufficient non-contract broiler farmers to participate in such a study may be challenging.

## 6. Conclusion

This paper describes antibiotic use practices in contract broiler farming in West Bengal through an agency theory lens. Currently, a decentralised production system reliant on small and medium-sized farmer enterprises remains reliant on antibiotics to mitigate risk and support production. Antibiotic alternatives, such as probiotics and dietary acidifiers, are beginning to replace antibiotics, but confidence of their efficacy is lacking in many settings. Open housing systems create challenging production environments with broilers being exposed to circulating infections and high temperatures and humidity. Those farmers seeking to upgrade their housing infrastructure, however, face barriers to accessing loans from financial institutions. Investigation of these barriers may provide insight into which mechanisms for infrastructural change will allow the sector to move away from antibiotics and towards alternatives. Stakeholder concerns around *Mycoplasma* in breeding stock results in the routine use of antibiotics belonging to classes deemed critically important for human health. This too requires further research to ascertain the validity of this concern and possible ways to mitigate this challenge without relying on antibiotics. Efforts to reduce antibiotic use in West Bengal’s broilers must not just focus on changing the prescribing behaviour of individuals but more broadly consider the environment within which broiler contracting exists.

## Supporting information

S1 File Interview topic guide for online interviews. (PDF)

S2 File Interview topic guide for fieldwork. (PDF)

S3 File Information sheet and consent form for online interviews. (PDF)

S4 File Information sheet and consent form for fieldwork. (PDF)

S5 File Checklist for inclusivity in global research. (DOCX)
