# Supplementary material for: Broiler farming and antibiotic use through an agency theory lens. A case study from West Bengal, India
Source: PLoS One. 2025 Jan 9;20(1):e0314090. doi: 10.1371/journal.pone.0314090 (PMC11717193; doi:10.1371/journal.pone.0314090)
Supplement: S1 File — (PDF) [file pone.0314090.s001.pdf]

Qualitative study into how inappropriate antibiotic use is defined, perceived and practiced, and the role of antibiotics in strategic decisions in broiler healthcare provision and acquisition

"Thank-you for taking the time to contribute to this research, I appreciate you are very busy – the interview should take one hour to complete.

Do you have any questions about the study or information sheet?

All of your data will be anonymised so that you cannot be identified. Do you give your consent for the interview to be audio recorded? Do you give consent for quotes from your interview to be used in study reports and publications?

Do you have any questions before we start?

Q0. Can you give me an overview of your experience of working with broiler poultry?

Part 1. General antibiotic use

1. What are your thoughts/views about the way antibiotics are used in broiler production?
    a. How has antibiotic use changed over time and why has this happened?
2. What reasons are antibiotics used in broiler production?
    a. Prompt – Are there reasons other than therapeutic use, prophylaxis and growth promotion? Are there any social or economic reasons why antibiotics may be used?
3. Who advises broiler producers about antibiotic use?
    a. Prompt – Where do people get information from about antibiotics?

Part 2. Strategic interactions

4. How do broiler producers choose which livestock healthcare provider to use?
    a. Prompt – What factors are involved and why - (e.g. costs of access, time, distance, familiarity, access to medicines)

5. When might disagreements occur between the broiler producers and livestock healthcare provider, how are these managed?
    b. Prompt - What happens if farmers are unhappy with the service provided to them? Would farmers and livestock healthcare provider ever disagree on the antibiotics given? Can you tell me of your experience/give an example of this?

6. How do the way antibiotics are used in the poultry industry affect how healthcare providers work and use antibiotics?
    c. Prompt – Do healthcare providers know what others, such as their rivals, are doing and do they change their behaviour accordingly? Why do they change behaviour? What are the consequences of not doing this? How do livestock healthcare providers remain competitive?
    d. Prompt – Do interactions with pharmaceutical reps affect how antibiotics are used?
7. How does the way antibiotics are used affect how broiler producers plan and operate their business?
    e. Prompt- do decisions around infrastructure, vaccines, production cycle change if antibiotic access changes?
    f. Prompt - Are broiler producers influenced by the way other producers use antibiotics?
    g. What type of contracts exist to stipulate how antibiotics are used?
    h. How have things changed since the colistin ban?
8. Who do you think has the control over strategies which dictate how antibiotics are used?
    a. Prompt – how are these decisions made?

Part 3. Misuse of antibiotics

9. How would you classify antibiotic misuse in broiler poultry?
    i. Prompt – can you give examples of antibiotics being misused, what criteria would you use to judge this?

j. Prompt – can you give examples of when other people have used antibiotics inappropriately? Why did this happen?

10. Can you give examples of antibiotics being used properly/rationally/appropriately?
11. What do you think is the extent of inappropriate* antibiotic use in boilers?
    a. – In the different type of systems

Part 4: Discussion of policy and guidelines

12. Who regulates antibiotic use in poultry?
    k. Prompt – which people or organisations have most power in regulating antibiotic use?
13. What type of guidelines are available for the use of antibiotics in poultry production?
    l. How do these guidelines influence antibiotic use?
    m. If guidelines exist how are these implemented and monitored? Are there any consequences for breaking these guidelines, or incentives for following them?

"The Indian National Action Plan on antimicrobial resistance recommends restricting and phasing out the use of antibiotics as growth promotion and prophylaxis in animals and restricting and phasing out the use of antibiotics in animals which are deemed critically important for humans – such as quinolones like enrofloxacin, and third and fourth generation cephalosporins."

14. How do you think these guidelines relate to poultry production?
15. What are the barriers to implementing these guidelines in broiler production and what effect would they have if they were fully enforced?
    a. Prompt – would businesses still be viable/able to make a profit?
    b. Prompt - How would people react about the restriction of antibiotics?

"Thank you for your time. Do you have any questions you would like to ask about the study?

Would you like to be provided with a copy of the study report, including a set of suggested guidelines for antibiotic use in broiler production, once the data has been analysed? If so, we will send this to you and provide you with the opportunity to comment on the findings.”
