# Supplementary material for: Broiler farming and antibiotic use through an agency theory lens. A case study from West Bengal, India
Source: PLoS One. 2025 Jan 9;20(1):e0314090. doi: 10.1371/journal.pone.0314090 (PMC11717193; doi:10.1371/journal.pone.0314090)
Supplement: S2 File — (PDF) [file pone.0314090.s002.pdf]

Broiler farming and antibiotic (mis)use in West Bengal

Topic guide for in-depth interviews

This guide serves as an overview for the type of questions which will be asked of participants. The semi-structured nature of the interviews allows for probing questions and novel lines of enquiry to be followed. In life history interviews, many of the questions will aim at understanding how their personal evolution within the industry and how this has led to the current farming situation. This will allow identification of the major strategic decisions taken over the years, and with this to understand the factors that have led to the current use of antimicrobials and potential future changes.

1. History
    a. What led people into their current roles/jobs?
    b. How do people think the broiler industry has changed over time?
    c. How their job has changed over time?
2. Access to inputs – focusing on how things have changed over time and why:
    a. Land – who owns what, how is land accessed?
    b. Poultry – dependency on certain companies, supply options, quality issues.
    c. Feed – dependency of supply, quality issues.
    d. Water – varying access to water, quality issues
    e. Power – how is the accessed and how reliable is the supply?
    f. Healthcare – who provides this and why, and how has this changed
    g. Medicines – who has agency over what medicines to give, where are medicines procured from?
    h. Who controls these inputs, what is the role of contract companies on these inputs?
    i. What type of regulation exists around inputs, how has this changed over time?
3. Wages and labour
    a. Role of the broiler industry in changing the livelihood of the participant and those people in the community
    b. Opportunity cost of broiler farming
    c. How broiler production affects debt and the accumulation of capital (financial and social)
4. Green revolution
    a. Challenges around intensive broiler production

        i. Climate and the suitability of broilers in India

   b. Changes in the way broilers are perceived as commodities
   c. Technology needed to support production
        i. Attitudes towards environmentally controlled housing
        ii. Supply chain infrastructure
   d. What's on the horizon – what change will happen in the next 5-10 years?

5. Antibiotic use
   a. How are antibiotics used?
   b. How has this changed, who has influenced change, why did it happen?
   c. What is the future of antibiotic use?
   d. What would be the consequences of changing antibiotic use?
   e. What regulations exist around antibiotic use and how do people interact with these?
