# Supplementary material for: Broiler farming and antibiotic use through an agency theory lens. A case study from West Bengal, India
Source: PLoS One. 2025 Jan 9;20(1):e0314090. doi: 10.1371/journal.pone.0314090 (PMC11717193; doi:10.1371/journal.pone.0314090)
Supplement: S3 File — (PDF) [file pone.0314090.s003.pdf]

This study has been approved by the Royal Veterinary College Ethical Committee (reference number: URN SR2021-0095)

Information sheet and consent form

Principle investigators: Mathew Hennessey (RVC PhD student), Pablo Alarcon (RVC PhD principal supervisor), Indranil Samanta (WBUAFS), Meenakshi Gauthman (LSHTM), Guillaume Fournie (RVC), Matthew Quaife (LSHTM), H. I. Paleja (Anand Agricultural University), Kumaravel Papaiyan (TANUVAS)

Current research: Investigating healthcare decision making and antibiotic use in India's broiler sector

Dear Sirs and Madams,

I am a PhD student working with a team of researchers from the Royal Veterinary College (London), the London School of Hygiene and Tropical Medicine (London), West Bengal University of Animal and Fishery Science, Anand Agricultural University (Gujarat), and Tamil Nadu Veterinary and Animal Sciences University. The research is also part of two ongoing research projects: OASIS – One Health Antibiotic Stewardship in Society and the One Health Poultry Hub.

My PhD is studying the type of healthcare decisions which are made in livestock production systems in India and the role medicines play in how these decisions are made. Over the next two months we will be interviewing people with experience of the broiler industry in India and people who provide healthcare services and medications including antibiotics.

We would very much appreciate your help and collaboration in this project. Using the results of this project we hope to be able to advise on how interventions to improve the use of medicines, including the policies governing antibiotics, could be developed in the poultry sector. We believe the results of this project would be useful for you and we would be happy to communicate them once they are ready.

We would like to inform you that any personal data (names, phone numbers, and email addresses) provided during the interview will remain confidential to the research team and will only be used for the purposes of this project. Your identity will remain anonymous in any reports or presentations. With your consent the interview will be recorded with a digital voice recorder (no video will be recorded). The voice recording will only be used for the purpose of this project and will only be accessible to the project team. Nevertheless, you have the right to stop the recording at any time during the interview and to request the erasure of anything that you do not want to be recorded. All the data collected will be analysed by the main researchers named above. Confidential data will be treated securely by being held in a password protected file stored on the RVC secure drive and not shared with anyone outside the immediate research team.

I would like to draw to your attention to the fact that any data that you provide to the researchers of this project, with the exception of personal or confidential information, could be published in form of scientific reports and/or in scientific papers; we may also use your words along with those of others to describe particular views or experiences relevant to the research. However, all personal data concerning you and your practice will be maintained anonymous at all times. Please note that providing your consent does not affect your right to stop this interview at any point.

This study has been approved by the Royal Veterinary College Ethical Committee (reference number: URN SR2021-0095**)**

If you have any questions regarding the study please contact:

Mathew Hennessey mphennessey@rvc.ac.uk

https://www.rvc.ac.uk/about/our-people/mat-hennessey

Pablo Alarcon palarcon@rvc.ac.uk

Participant consent:

For interviews taking place online, we will explain conditions for the interview and then ask you to provide your verbal consent before the interview starts.

If you prefer, you can complete the following section and return it to mphennessey@rvc.ac.uk before the interview.

"I consent to the interview being audio recorded and quotes from the interview used for internal reports within the project partners institutions and in documents that will be in the public domain such as external reports and published scientific research papers".

Name: ______________________________

Date: _______________________________

Signature: ___________________________

Please indicate whether you would like to receive a copy of the study report and an opportunity to comment on the findings:

☐ **Yes, I would like to receive a copy of the study report**

Please provide an email address where the report can be sent:

____

☐ **No, I would not like to receive a copy of the study report**
