# Supplementary material for: Broiler farming and antibiotic use through an agency theory lens. A case study from West Bengal, India
Source: PLoS One. 2025 Jan 9;20(1):e0314090. doi: 10.1371/journal.pone.0314090 (PMC11717193; doi:10.1371/journal.pone.0314090)
Supplement: S4 File — (PDF) [file pone.0314090.s004.pdf]

This study has been approved by the Royal Veterinary College Ethical Committee (reference number: URN SR2021-0196)

Information sheet and consent form – participant observation

Principle investigators: Mathew Hennessey (RVC PhD student), Pablo Alarcon (RVC PhD principal supervisor), Indranil Samanta (WBUAFS), Meenakshi Gauthman (LSHTM), Guillaume Fournie (RVC), Matthew Quaife (LSHTM)

Investigating healthcare decision making and antibiotic use in India's broiler sector

Dear Sirs and Madams,

I am a PhD student working with a team of researchers from the Royal Veterinary College (London), the London School of Hygiene and Tropical Medicine (London) and West Bengal University of Animal and Fishery Science. The research is also part of an ongoing research project in West Bengal: OASIS – One Health Antibiotic Stewardship in Society.

My PhD is studying the type of decisions which are made in broiler production systems in India and the role medicines play in how these decisions are made. Over the next two months we will be spending time with people who work in the broiler industry in West Bengal. During this time, we will be observing the types of activities which people participate in when raising broilers and talk to people about these activities. If you provide your consent to do so, we may take notes, voice recordings, photos and video to document these observations.

We would very much appreciate your help and collaboration in this project. Using the results of this project we hope to be able to advise on how interventions to improve the use of medicines, including the policies governing antibiotics, could be developed in the poultry sector. We believe the results of this project would be useful for you and we would be happy to communicate them once they are ready.

We would like to inform you that any personal data (names, places, institutions, phone numbers, and email addresses) provided during the study will remain confidential to the research team and will only be used for the purposes of this project. Your identity will remain anonymous in any reports or presentations.

With your consent, written fieldnotes, audio recording, photographs and video recordings will be taken to document parts of the broiler production system. Fieldnotes and data from audio recordings may be used for both internal reports accessible to the project team and external reports which may be available in the public domain. Visual material – photos and video – which could be used to identify yourself or your business will only be made available to the project team, and will not be used in external reports or presentations. Photographs which do not allow the identification of people of businesses may be used in reports which could enter the public domain.

You have the right to request the erasure of anything that you do not want to be kept recorded. All the data collected will be analysed by the main researchers named above. Confidential data will be treated securely by being held in a password protected file stored on the RVC secure drive and not shared with anyone outside the immediate research team.

I would like to draw to your attention to the fact that any data that you provide to the researchers of this project, with the exception of personal or confidential information, could be published in

This study has been approved by the Royal Veterinary College Ethical Committee (reference number: URN SR2021-0196)

form of scientific reports and/or in scientific papers; we may also use your words along with those of others to describe particular views or experiences relevant to the research.

All personal data concerning you will be kept anonymous at all times. Please note that providing your consent does not affect your right to stop the observational study at any point.

If you have any questions regarding the study, please contact:

Mathew Hennessey mphennessey@rvc.ac.uk, https://www.rvc.ac.uk/about/our-people/mat-hennessey

Pablo Alarcon palarcon@rvc.ac.uk

Participant consent:

"I have been provided with information about what my participation in this study involves and I have been given the opportunity to ask questions". ☐

"I consent to field notes and audio recordings being taken and this data used for internal reports within the project partners institutions and in documents that will be in the public domain such as external reports and published scientific research papers". ☐

"I consent to photographs and video recordings being taken. Photographs will only be used in reports and publications if they do not allow the identification of people or businesses." ☐

Name: ______________________________

Date: _______________________________

Signature: __________________________

Please indicate whether you would like to receive a copy of the study report:

☐ **Yes, I would like to receive a copy of the study report**

Please provide an email address where the report can be sent:

____
